# Epidemiology and factors associated with the infection of *Babesia bigemina*,* Babesia bovis*, and *Theileria orientalis* in Thale Noi Wetland buffaloes (*Bubalus bubalis*), Southern Thailand

**DOI:** 10.1186/s12917-025-04846-1

**Published:** 2025-06-02

**Authors:** Chalutwan Sansamur, Kanpapat Boonchuay, Ruttayaporn Ngasaman, Kenny Oriel Aranas Olana, Veerasak Punyapornwithaya

**Affiliations:** 1 Akkhararatchakumari Veterinary College, Walailak University, Nakhon Si Thammarat, Thailand; 2 Centre for One Health, Walailak University, Nakhon Si Thammarat, Thailand; 3 Faculty of Veterinary Science, Prince of Songkla University, Songkhla, Thailand; 4 Department of Veterinary Paraclinical Sciences, College of Veterinary Medicine, Visayas State University, Baybay City, Philippines; 5 Research Center for Veterinary Biosciences and Veterinary Public Health, Faculty of Veterinary Medicine, Chiang Mai University, Chiang Mai, Thailand

**Keywords:** Water buffalo, *Babesia bovis*, *Babesia bigemina*, *Theileria oreintalis*, Risk factors, Multifaceted farming system

## Abstract

**Background:**

Water buffaloes (*Bubalus bubalis*) play a critical role in maintaining biodiversity and supporting the local economy within the unique ecosystem of the Thale Noi Wetlands in Southern Thailand, a globally recognized heritage site. However, these buffaloes are commonly infected by hemoprotozoan parasites, which can lead to severe pathological conditions. This study aims to investigate the epidemiology of hemoprotozoan infections, assess the occurrence of co-infections among these pathogens, and identify the risk factors associated with infections in water buffalo. A total of 155 water buffaloes from 43 farms in the Thale Noi Wetlands were included in the study, and their blood samples were analyzed using PCR to detect the presence of *Babesia bigemina*, *Babesia bovis*, and *Theileria orientalis*. A phylogenetic analysis based on the *T. orientalis* gene encoding a major piroplasm surface protein was performed to assess genetic diversity. Additionally, questionnaire surveys were conducted on these farms. The associations between the presence of hemoprotozoan parasites and various risk factors were analyzed using logistic regression. Furthermore, Multiple Correspondence Analysis (MCA) was employed to explore the patterns and relationships among single and co-infections and associated factors.

**Results:**

The prevalence of *T. orientalis*, *B*. *bigemina*, and *B*. *bovis* infections were 29.37**%**, 23.81**%**, and 5.6**%**, respectively, with triple infections accounting for 4.76**%.** The *T. orientalis* genotype N-1 was identified as the predominant genotype. The major risk factors for *T. orientalis* infection included poor body condition and younger buffaloes **(**1–5 years old**).** Male buffaloes had higher odds of *B*. *bovis* infection than female buffaloes. Additionally, the MCA identified significant clustering of single infections and co-infections, with *T*. *orientalis* and *B*. *bigemina* co-infection showing the strongest association and emerging as the most prevalent **(**25.39**%).** Younger buffaloes and those with poor body condition scores had higher odds of co-infections.

**Conclusion:**

This study reveals a high prevalence of hemoprotozoan infections among water buffaloes in the Thale Noi Wetland. Co-infection with *T. orientalis* and *B. bigemina* emerged as a significant finding. Additionally, the *T. orientalis* Type N-1 strain was identified as the predominant genotype, representing the first documented report of its presence in Southern Thailand. Younger age and poor body condition were key risk factors associated with infection. Addressing these factors through targeted interventions may enhance buffalo health and productivity within this ecologically important ecosystem.

**Supplementary Information:**

The online version contains supplementary material available at 10.1186/s12917-025-04846-1.

## Background

Piroplasmosis in ruminants, including babesiosis and theileriosis, has garnered considerable attention from researchers due to its impact on global cattle production and potential public health implications [1–5]. Moreover, the infestation of ectoparasites further compounds the health challenges, resulting in significant tick burdens and clinical manifestations of bovine babesiosis and theileriosis. The principal agents responsible for bovine babesiosis are *Babesia bigemina* and *Babesia bovis* [6, 7]. Clinical features of *Babesia* spp. infection typically encompasses high fever, anorexia, and hemoglobinuria [8]. *Theileria orientalis* is a causative agent of benign theileriosis in bovines and is distributed mainly in Southeast Asia [9–12]. Infected ruminants may experience hemolytic anemia, jaundice, and abortions [13].

Water buffaloes (*Bubalus bubalis*), belonging to the family *Bovidae* and sub-family *Bovinae*, have both agricultural and economic significance in various countries, including Thailand [14, 15]. However, buffaloes often suffer from ectoparasites infestations that act as vectors for hemoprotozoan parasites such as *Babesia* spp. and *T. orientalis*. Such parasites are predominantly transmitted through tick bites, affecting both animal and human hosts, remaining carriers of these pathogens, representing a risk of infection for more susceptible species like cattle [16]. Specifically, *B. bigemina* and *B. bovis* are primarily transmitted by the one-host tick *Rhipicephalus* (*Boophilus*) *microplus* [17]. In contrast, *T. orientalis* is typically vectored by *Haemaphysalis* spp. [18]. However, previous studies in northern and northeastern Thailand reported that ticks collected and identified in those regions were exclusively *R. microplus* [19].

Incidences of bovine babesiosis and benign theileriosis have been reported in various parts of Thailand through multiple studies. Despite the documented impact of bovine babesiosis and benign theileriosis in Thailand, epidemiological studies have primarily focused on dairy and beef cattle populations [19–22]. Consequently, there is limited information on the epidemiology and risk factors of these parasites in water buffaloes, particularly in Southern Thailand [2, 23, 24]. Given that water buffaloes constitute a reservoir for hemoprotozoan parasites, infections are likely to remain undetected during the subclinical or chronic stages, especially in the presence of co-infections [2]. Molecular diagnostic techniques based on nucleic acid amplification, such as polymerase chain reaction **(**PCR**)** and nested PCR (nPCR), have proven to be highly effective in detecting such pathogens. These approaches offer markedly greater analytical sensitivity and specificity compared to conventional diagnostic methods, such as Giemsa-stained blood smear examination [21, 25].

The Thale Noi buffalo is a distinct breed originating from the Thale Noi Wetlands [26, 27]. Thale Noi, a prominent natural lake in Southeast Asia, has been formally recognized as a Globally Important Agricultural Heritage System (GIAHS), emphasizing the significance of biodiversity conservation and sustainable resource management. The Thale Noi Wetland Buffalo Pastoral Agro-Ecosystem represents a complex farming system, highlighting the long-standing interaction between humans and buffaloes [28]. Like other livestock populations, they are vulnerable to various infectious diseases that can significantly affect their health, productivity, and economic value. The spread of hemoprotozoan parasites poses a particular threat, as these infections can lead to substantial health issues if not properly managed. A comprehensive understanding of the epidemiology of hemoprotozoan parasites is crucial for assessing the vulnerabilities and health risks associated with these diseases in this unique ecosystem.

This study aims to assess the prevalence of hemoprotozoan parasites in water buffalo populations and identify the associated risk factors at the individual animal level. It provides epidemiological information on the burden of disease and serves as a useful guide for future interventions to improve buffalo health.

## Results

### General characteristics of animals and farm practices

In this study, 155 water buffaloes from 43 farms located in 26 different areas within the Thale Noi Wetlands were sampled and examined for hemoprotozoan parasites. A total of 43 respondents agreed to participate in the study and were successfully interviewed. The majority of respondents were male (79.07%, *n* = 34), under the age of 50, and had attained an education up to the primary school level. Regarding water buffalo rearing, most farmers (67.44%, *n* = 29) reported owning five or more animals per household with the primary purpose of earning extra income through their sale (72.09%, *n* = 31). A smaller proportion of farmers focused specifically on breeding buffaloes (4.65%, *n* = 2), while 23.26% (*n* = 10) pursued both objectives. In terms of farm management practices, 41.86% (*n* = 18) of the farmers followed a combination of stall feeding and free grazing, whereas 58.14% (*n* = 25) exclusively practiced free grazing during the daytime. Additionally, 60.47% of farmers preferred to raise buffaloes in communal herds rather than individually (*n* = 26). It was also observed that 69.77% of farmers had a few beef cattle on their farms (*n* = 30), while 23.26% purchased new animals without adhering to quarantine protocols (*n* = 10). Notably, all farmers used bulls for breeding purposes. Finally, only 34.88% (*n* = 15) of the farmers had received training on buffalo management and ectoparasite control programs.

Out of the 155 buffaloes sampled, 60% were male (*n* = 93) and 40% female (*n* = 62). Regarding age distribution, 38.06% of the animals were aged > 5 years (*n* = 59), and 61.93% were 1–5 years old (*n* = 96). Ectoparasites were present in 20.64*% (n* = 32*)* of the animals. Nearly half were found to be in poor condition. Specifically, the body condition scoring (BCS) indicated that 45.16*% (n* = 70*)* were categorized as thin and emaciation, while 54.84% were in good condition. Additionally, 34.88% of the farmers reported a history of abortion in the previous 12 months.

**Fig. 1:**
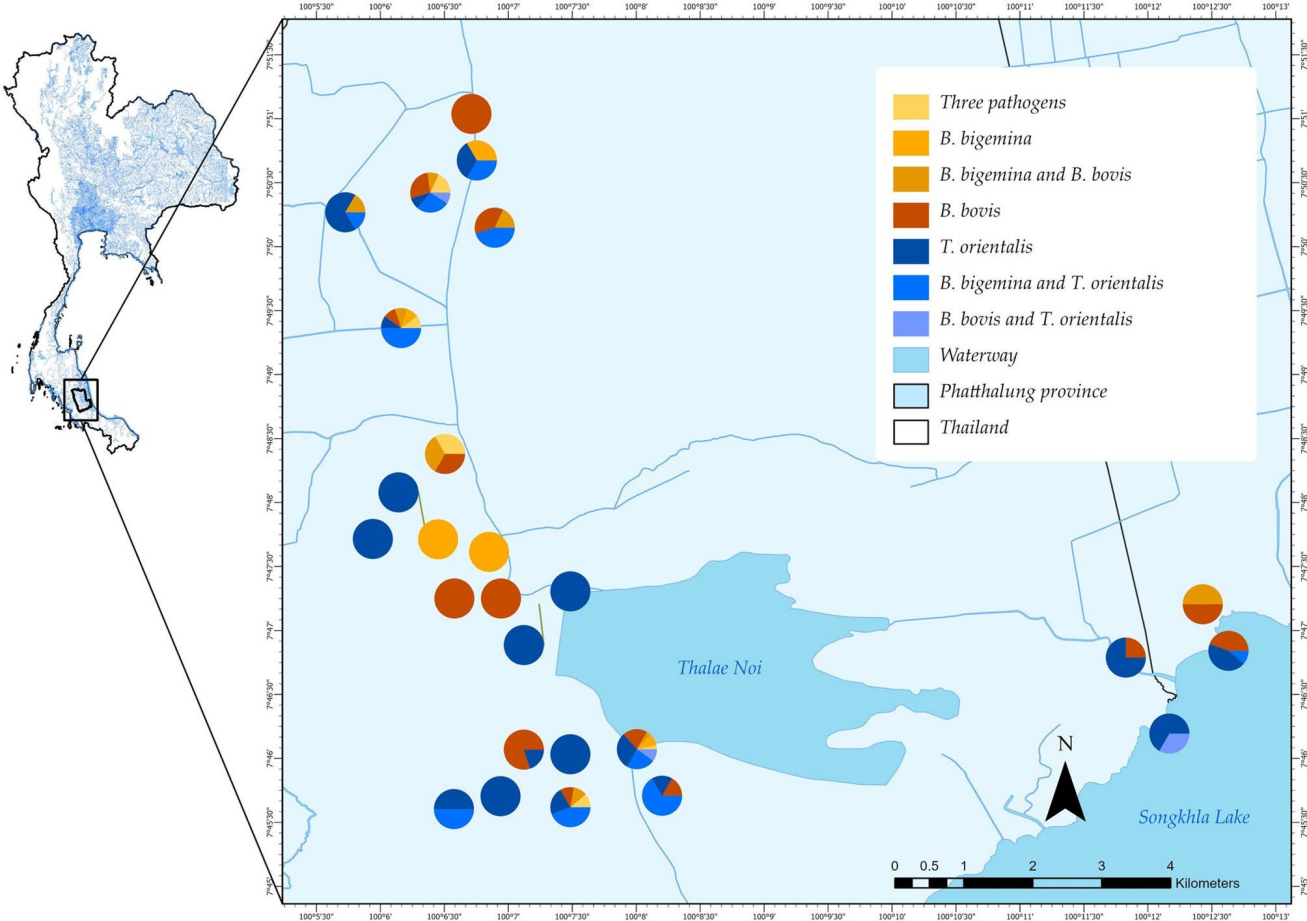
Distribution map of single and multiple blood parasite infections among water buffalo farms in the Thale Noi Wetlands, Phatthalung Province, Southern Thailand. The map was generated using QGIS open-source software available at http://qgis.osgeo.org

Detailed information on hemoprotozoan parasite infection is summarized in Table 1 and illustrated in Fig. 1. Of the 155 water buffaloes examined, 126 were found to be infected. The prevalence of single infection of *T. orientalis*, *B. bigemina*, and *B. bovis* in water buffaloes were 29.36% (*n* = 37/126), 23.81% (*n* = 30/126), and 5.6% (*n* = 7/126), respectively. Furthermore, triple infections accounted for 4.76% (*n* = 6/126) of the total. Among the co-infections, the highest prevalence was observed between *B. bigemina* and *T. orientalis* (25.39%, *n* = 32/126). This was followed by co-infections between *B. bigemina* and *B. bovis* (7.14%, *n* = 9/126) and those between *B. bovis* and *T. orientalis* (3.97%, *n* = 5/126).

**Table 1:** Single and co-infections in water buffaloes

Parasite infection	Frequency (*N* = 126)*	Prevalence (95%CI)**
**One pathogen**		
*T. orientalis*	37	29.36 (22.71–36.79)
*B. bigemina*	30	23.81 (17.23–31.45)
*B. bovis*	7	5.6 (2.79–9.61)
**Two pathogens**		
*B. bigemina* and *T. orientalis*	32	25.39 (18.60–33.65)
*B. bigemina* and *B. bovis*	9	7.14 (3.80–13.02)
*B. bovis* and *T. orientalis*	5	3.97 (1.71–8.95)
**Three pathogens**		
*B. bigemina*, *B. bovis*, and *T. orientalis*	6	4.76 (2.20–10.0)

*Total of infection**95% confidence interval

**Fig. 2:**
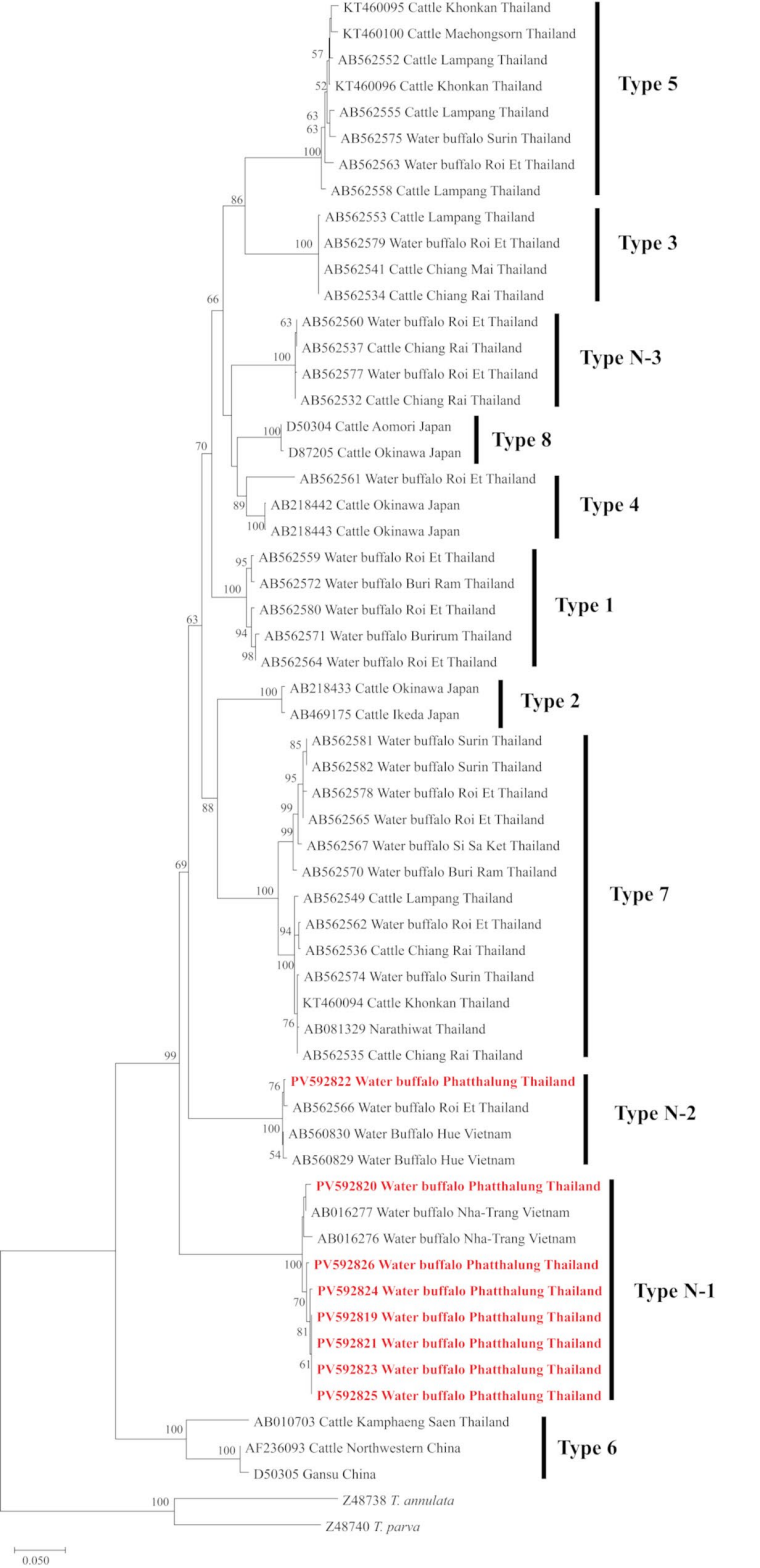
A phylogenetic tree of *T. orientalis* MPSP gene sequences derived from water buffaloes in Southern Thailand (eight sequences; GenBank accession numbers PV592819-PV592826), along with previously registered sequences from the GenBank database (51 sequences), was constructed. The sequences obtained in this study are highlighted in bold for clarity. Vertical bars indicate the 11 recognized genotypes of *T. orientalis* lineages, as described by Altangerel et al. (2011) [23]. Bootstrap values greater than 50% are displayed at the branch points to indicate the reliability of the clustering

In the phylogenetic analysis, the *T. orientalis* sequences obtained in this study grouped into two distinct clades, genotypes N-1 and N-2. Genotype N-1 was the predominant genotype detected in water buffaloes in this study. The homology of this clade was 99.85–99.96%, indicating a degree of genetic variation within this genotype. This genotype was characterized based on phylogenetic comparison with previously reported *MPSP* gene sequences, including those documented in Vietnamese *T. orientalis* (AB016267 and AB016277). In contrast, only a single sequence was assigned to genotype N-2, exhibiting 99.94–99.97% similarity to reference sequences within this clade, indicating relatively low genetic divergence. Interestingly, this is consistent with the previously reported Roi Et region Thailand genotype (AB562566), which also showed high similarity within this clade (Fig. 2.).

### Analysis of risk factors for the presence of *B. bigemina*, *B. bovis*, and *T. orientalis*

For *B. bigemina*, in the univariable logistic regression analysis, poor BCS was associated with a higher likelihood of the presence of *B. bigemina*; however, no variables were found to be significantly associated with *B. bigemina* in the multivariable logistic regression model. Sex was a significant factor for *B. bovis*, with males showing a higher likelihood of the presence of *B. bovis* in both the univariable and multivariable logistic regression models (OR = 3.25, 95%CI = 1.39–7.93). In relation to *T. orientalis*, univariable logistic regression identified age and BCS as significant factors. In the multivariable model, younger water buffaloes (1–5 years) (OR = 2.85, 95%CI = 1.37–6.11) and those with a thin body condition (OR = 2.49, 95%CI = 1.75–3.63) were at a higher risk of the presence of *T. orientalis* (*p* < 0.05). Further details are provided in Tables 2 and 3.

**Table 2:** Factors associated with the presence of *Babesia bigemina*, *Babesia bovis*, and *Theileria orientalis* based on univariable logistic regression analysis at the animal level

Variable	Categories	Control	Case	*p*-value	OR	95%CI*
***Babesia bigemina***						
Sex	Female	31	30			
	Male	47	47	0.92	0.96	0.48–1.93
Age (years)	> 5 years old	31	28			
	1–5 years old	47	49	0.66	1.15	0.57–2.32
Body condition status	Good	38	47			
	Poor	40	30	0.12	0.6	0.3–1.2
Ectoparasites infestation	No	63	60			
	Yes	15	17	0.66	1.19	0.55–2.62
***Babesia bovis***						
Sex	Female	44	17			
	Male	84	10	0.005	3.21	1.26–8.59
Age (years)	> 5 years old	31	28			
	1–5 years old	47	49	0.45	0.72	0.27–1.61
Body condition status	Good	71	14			
	Poor	57	13	0.73	1.15	0.28–1.85
Ectoparasites infestation	No	102	21			
	Yes	26	6	0.82	1.12	0.38–2.92
***Theileria orientalis***						
Sex	Female	42	52			
	Male	33	28	0.25	0.68	0.34 - 1.37
Age (years)	> 5 years old	31	13			
	1–5 years old	40	60	< 0.001	3.22	1.56–6.8
Body condition status	Good	58	27			
	Poor	17	53	< 0.001	6.6	3.11–14.58
Ectoparasites infestation	No	57	66			
	Yes	18	14	0.31	0.67	0.34–1.33

**Table 3:** Factors associated with the presence of *Babesia bovis* and *Theileria orientalis* at the animal level based on multivariable logistic regression

Variable	Categories	Control	Case	*p*-value	Adjusted OR	95%CI
***Babesia bovis***						
Sex	Female	44	17			
	Male	84	10	0.007	3.25	1.39–7.93
***Theileria orientalis***						
Age (years)	> 5 years old	31	13			
	1–5 years old	40	60	0.006	2.85	1.37–6.11
Body condition status	Good	25	20			
	Poor	17	53	< 0.001	2.49	1.75–3.63

Hosmer and Lemeshow test *p*-value = 0.91

### Co-infections of *Babesia bigemina*, *Babesia bovis*, and *Theileria orientalis *and risk factors

**Fig. 3:**
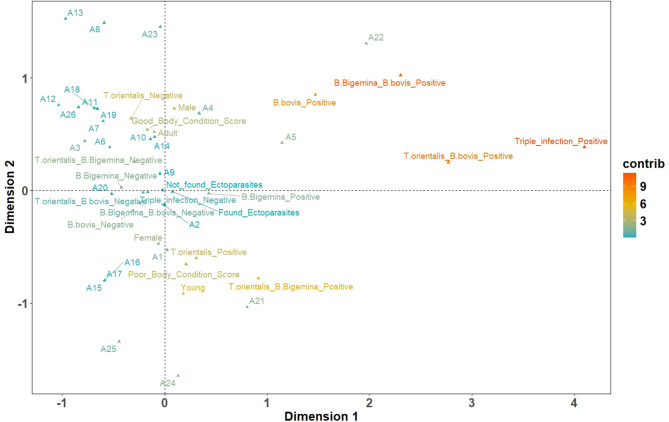
The multiple correspondence analysis map presents the relationships between the presence of *B. bigemina*, *B. bovis*, and *T. orientalis* (single infection and co-infection) and specific variables of water buffaloes under study, including age, communal herd location, sex, ectoparasite infestation, and body condition scores. Blue indicates low contribution, yellow moderate contribution, and red high contribution (contrib)

In this analysis, the first two Multiple Correspondence Analysis **(**MCA**)** dimensions explained a cumulative variance of 16.16%, with Dimensions 1 and 2 accounting for 9.04% and 7.12% of the total inertia, respectively (Fig. 3.). The results revealed that single infections by *B. bigemina*, *B. bovis*, and *T. orientalis* followed common infection patterns, as these pathogens clustered closely along the primary dimension, suggesting the influence of shared underlying factors. Furthermore, co-infections involving two pathogens demonstrated a distinct and consistent contribution to inertia, indicating a structured relationship between the pathogens. Among these, the co-infection of *T. orientalis* and *B. bigemina* (0.762) exhibited the strongest association, followed closely by *T. orientalis* and *B. bovis* (0.701) and *B. bovis* and *B. bigemina* (0.686).

Younger animals exhibited a significant contribution, as evidenced in Dimension 2 (11.85%). Similarly, BCS played a substantial role in infection susceptibility, with poor BCS being linked to higher contributions to Dimension 2 (35.3%). This finding indicated that younger and poor BCS buffaloes were at greater risk of co-infection. Additionally, sex differences were also identified, with males exhibiting a higher risk of co-infection compared to females. Although ectoparasite infestation and location variables demonstrated minimal overall influence on the relationship between infections and co-infections, their potential contributions should not be overlooked. When included as categorical variables in the MCA, their associations with specific infection types would be discernible through the spatial distribution of their categories on the factor map.

## Discussion

This study determined the prevalence, co-infection patterns, and associated risk factors of hemoprotozoan parasites in water buffaloes within the Thale Noi Wetlands. To the best of our knowledge, this study represents the first comprehensive investigation into the epidemiology and risk factors associated with *B. bigemina*, *B. bovis*, and *T. orientalis* infections in water buffaloes from Southern Thailand. In the present study, *T. orientalis* and *B. bigemina* exhibited a higher prevalence compared to *B. bovis*, aligning with that reported in Northeastern Thailand, of 3.6%, 11.2%, and 25% for *B. bigemina*, *B. bovis*, and *T. orientalis*, respectively [2, 23]. A similar pattern was noted in Egypt [29, 30], Colombia [31], and several Asian countries like Vietnam [32, 33], South China [4] and Sri Lanka [3, 9]. In contrast, high prevalence was close to 50% in Argentina [3], Western Cuba [34], and Brazil [1, 35]. The variability in prevalence across different geographical regions may be attributed to divergences in tick distribution, tick vector infection, vector-pathogen interactions, climate patterns, livestock breeds, farm management practices, and sample collection [9, 36, 37]. Phylogenetic analysis of the *T. orientalis* MPSP gene sequences obtained in this study revealed the presence of two distinct clades: Type N-1 and Type N-2. Notably, this study is the first to report the presence of Type N-1 in Southern Thailand, highlighting its unique genetic signature in this region. Prior to this study, seven genotypes (Type 1, 3, 4, 5, 7, N-2, and N-3) were documented exclusively in the northeastern part of Thailand [23]. Interestingly, both Type N-1 and Type N-2 had been previously identified in *T. orientalis* populations in Vietnam [38]. These findings not only expand the known geographic distribution of *T. orientalis* genotypes in Thailand but also highlight the need for ongoing molecular surveillance to monitor the emergence and spread of new genotypes.

In this study, all sampled water buffaloes had at least one hemoprotozoan infection. Among these, the presence of co-infections of *B. bigemina* and *T. orientalis* (25.39%) emerged as the most prevalent, determined by polymerase chain reaction (PCR) detection, and were identified as the dominant contributors in the MCA (0.762). These results suggest that *T. orientalis* plays a “bridge role” in co-infections, often appearing in conjunction with other pathogens. For instance, the term “piroplasms” refers to these parasites’ shared morphology, pear-shaped forms within erythrocytes consistent across regions [6]. While *T. orientalis* is typically characterized as a benign theileriosis, interaction with other co-infecting parasites in different geographical and ecological contexts may exacerbate its pathogenic potential [12]. This bridge role may be explained by shared vectors. Both *Babesia spp.* and *T. orientalis* are transmitted by *Rhipicephalus microplus*, while *T. orientalis* can also be mechanically transmitted by tabanid flies (*Tabanus megalops*) [20]. Such overlapping vector exposure increases the risk of co-infections. Although the immunological interplay between pathogens is not fully understood, chronic infection with one parasite may influence the host’s susceptibility to others. These dynamics emphasize the need to consider host–vector–pathogen interactions when addressing co-infections in livestock [34, 39].

In this study, age and BCS were identified as key host characteristics associated with the single and co-infection status of *T. orientalis* after being analyzed using the multiple logistic regression model and MCA, respectively. The major risk factors for infection with *T. orientalis* included younger water buffaloes (1–5 years) (OR = 2.85, 95%CI = 1.37–6.11) and poor BCS (OR = 2.49, 95%CI = 1.75–3.63). Furthermore, the MCA map showed that co-infections of *T. orientalis* and *B. bigemina* contributed significantly to Dimension 1, while younger animals and poor BCS were closely associated with Dimension 2. Consistent with our findings, previous studies have reported a significantly higher prevalence of *T. orientalis* infection in younger buffaloes [40, 41], which were also more likely to present with poor BCS [40]. The significantly higher infection rate observed in buffaloes under five years of age may be attributed to the development of acquired immunity in older animals, enabling them to maintain resistance and reduce their susceptibility to recurrent infections. Acquired immunity plays a pivotal role in achieving enzootic stability, as exposure to pathogens over time allows animals to develop protective immunity, ultimately resulting in a well-protected adult herd [42]. In the Thale Noi ecosystem, where free-grazing buffaloes from mixed herds roam across extensive wetland pastures, the risk of encountering ticks and other vectors is elevated. These conditions facilitate early and repeated vector exposure among young animals before they can mount effective immune responses. Although a few studies report that older cattle in endemic areas develop a high degree of resistance to *Theileria* infection, they may still serve as carriers, acting as reservoirs for ticks and transmitting the infection to other cattle [11, 43].

This study revealed a correlation between male water buffaloes and *B. bovis* infection, while sex was not significantly associated with the presence of *T. orientalis* and *B. bigemina*. A study conducted in Egypt similarly found no correlation between gender and *Theileria* infection [44]. A higher prevalence of *B. bovis* in males compared to females has also been reported [45]. Moreover, tick infestation was not identified as a significant risk factor for any of the three pathogens, consistent with the MCA results. This may be due to the behavior of water buffaloes, which spend a substantial amount of time submerged in mud, likely reducing tick attachment and subsequent pathogen transmission [2]. However, in mixed farming systems, where infected buffaloes and ticks coexist, the risk of infection may persist as the transmission cycle continues [46, 47].

Based on the MCA and epidemiological results, host- and management-related factors influencing infection dynamics and co-prevalence were identified. Co-infections involving all three pathogens within a single host were observed. These co-infections amplified the clinical impact on infected animals, as suggested in earlier research relating to tick-borne diseases in ruminants [34]. The contribution of these co-infections to total inertia in the MCA suggests that pathogen co-occurrence may be driven by factors such as host susceptibility and vector competence. Additionally, the ecological overlap of vectors and shared transmission routes likely play a role in facilitating co-infections. These findings provide a basis for designing targeted interventions to mitigate the impact of these parasites on livestock health and productivity.

Traditional livestock management and botanical knowledge play a vital role in maintaining animal health in the Thale Noi Wetlands. Local farmers rely on indigenous practices and natural remedies to manage health issues, including hemoprotozoan diseases. While these methods offer valuable insights, they may lack consistent efficacy. Therefore, engaging farmers with veterinary approaches can help enhance their knowledge, particularly in disease prevention and control, improving livestock health management.

This study has several limitations. The identified risk factors were assessed at the individual animal level, limiting the ability to explain herd-level risk factors. Future studies should include a larger number of farms to provide more comprehensive insights. Despite these limitations, this study is the first to address the burden of *B. bigemina*, *B. bovis*, and *T. orientalis* infections, along with their associated risk factors, in animals from the areas under study. In addition, recent studies have identified other vector-borne pathogens in Thai water buffaloes, including *Anaplasma* spp., *Plasmodium* spp., and other piroplasms [22, 48]. Several of these have been reported in the same geographical area as the current study. Although this study focused specifically on *Babesia* spp. and *T. orientalis*, the potential co-circulation of additional hemoparasites further illustrates the spatial heterogeneity of pathogen distribution in buffalo populations. This highlights the complexity of host–vector–pathogen interactions across different ecological zones. Expanding future surveillance to include a broader spectrum of pathogens may enhance the understanding of co-infection dynamics and spatial epidemiological patterns, supporting more targeted and integrated disease control strategies.

## Conclusions

This study investigated the prevalence, co-infection patterns, and risk factors associated with hemoprotozoan parasites in water buffaloes within the Thale Noi Wetland. A key finding was the co-infection of *T. orientalis* and *B. bigemina*. Additionally, the study revealed notable genetic diversity of *T. orientalis* and is the first to report the presence of the Type N-1 strain in Southern Thailand, highlighting the need for continued surveillance in the region. The observed association between younger age, poor body condition scores, and increased infection risk further emphasizes the importance of targeted control measures to enhance buffalo health in this unique agro-ecosystem.

## Methods

### Study location and period

A cross-sectional survey was conducted from July to October 2021 within the Thale Noi Wetlands in Phatthalung Province, Southern Thailand, located at 7°46’00” N and 100°09’11” E. Thale Noi is one of Southeast Asia’s largest natural lakes, known for its rich ecological diversity and as a habitat for approximately 4,000 water buffaloes. This ecosystem, supported by abundant forage resources, provides an ideal environment for promoting the health and productivity of the buffalo population [25].

### Sampling and data collection

The sample size was calculated based on an estimated population of 4,000 water buffaloes using the Epitools program (https://epitools.ausvet.com.au/prevalencess). Parameters included a 95% confidence interval (CI), 0.1 precision, and prevalence data for *B. bigemina*, *B. bovis*, and *T. orientalis* from prior studies [2, 29] conducted in Thailand. A total of 155 water buffaloes were randomly selected from 43 farms in the Thale Noi Wetlands, Phatthalung Province. Simple random sampling was applied by generating a list of eligible buffaloes from each farm in collaboration with local livestock officers and farm owners. Individual animals were then selected using random number tables to avoid selection bias and ensure that each animal had an equal chance of inclusion. Blood samples were collected from each animal. The blood samples were aseptically obtained from the jugular vein and immediately transferred into 10-ml vacuum blood collection tubes containing an anticoagulant agent. Samples were placed in an ice box until their arrival at the Research Institute for Health Sciences (RIHS) laboratory at Walailak University, where they were stored at 4 °C. For each sampled animal, individual details such as age, sex, and health status were recorded. The study also assessed whether the buffaloes were kept with other animals or in isolation. A body condition scoring (BCS) system was applied to assess the buffaloes’ physical condition, categorizing them into two main groups: BCS 1–2 for poor condition and BCS 3–5 for good condition. Buffaloes in poor condition displayed extreme leanness, with prominent dorsal spines that were easy to feel and visible transverse processes with little to no flesh cover. In contrast, buffaloes in good condition had a noticeable fat layer covering the ribs, and their dorsal spines and transverse processes were either partially or fully covered by flesh, indicating adequate body reserves [49, 50].

During sample collection, an epidemiological questionnaire was used to gather information in three key areas: (1) general information about farmers, (2) animal-related information, and (3) farm-related information. Precise geographical coordinates (X and Y) were recorded for each farm visited. Before the final survey, a preliminary survey involving 15 participants was conducted to validate and ensure the reliability of the questionnaire. Both qualitative and quantitative data were carefully documented. A summary of the questionnaire content is presented in Supplementary Material 1.

### PCR detection of *T. orientalis*, *B. bigemina* and *B. bovis*

Genomic DNA was extracted from whole blood samples using the gSYNCTM DNA Extraction Kit (Geneaid Biotech Ltd., New Taipei City, Taipei), following the manufacturer’s instructions. The extracted DNA was stored at − 30 °C for subsequent molecular analysis. A PCR-based survey was then conducted to detect hemoprotozoan parasites, including *T*. *orientalis*, *B*. *bigemina*, and *B*. *bovis*, in water buffaloes in Thailand. The oligonucleotide primers and PCR assays used in this study are summarized in Table 4. Detection of *T. orientalis* was carried out using a single PCR assay targeting a 776-bp fragment of the major piroplasm surface protein *(MPSP*) gene, employing specific primers as described by Ota et al. (2009) [51]. In contrast, screening for *B. bigemina* and *B. bovis* was performed using nested PCR assays targeting the rhoptry-associated protein (RAP)-1a gene and the spherical body protein (SBP)-2 gene, respectively, based on the protocols of Terkawi et al. (2011) [2] and Aboulaila et al. (2010) [52]. Briefly, *T*. *orientalis* identification involved a 10 µl reaction mixture containing 1 µl of template DNA, 5 µl of 2X-KAPA2G Fast HotStart ReadyMix PCR kit^®^**(**Kapa Biosystems, Japan**)**, 1 pmol of each primer, and 2 µl of distilled water. The PCR protocol included an initial denaturation at 94 °C for 5 min, followed by 35 cycles of denaturation at 94 °C for 45 s, annealing at 58 °C for 1 min, extension at 72 °C for 1 min, and a final extension at 72 °C for 7 min. For *B*. *bigemina* and *B*. *bovis* parasite screening, nPCR was performed. The final volume of 10 µl contained 1 µl of template DNA and 9 µl of reaction mixture with 2X-KAPA2G Fast HotStart ReadyMix PCR kit^®^**(**Kapa Biosystems, Japan**)**, 1 pmol of each primer and topped up with distilled water to the final volume. The amplification conditions included an initial denaturation at 95 °C for 5 min, followed by 35 cycles of denaturation at 94 °C for 1 min, annealing at 64 °C for *B*. *bovis* and 55 °C for *B*. *bigemina* for 1 min, extension at 72 °C for 1 min, and final extension at 72 °C for 7 min. The same concentration of buffer and PCR master mix used for amplification of the 1 µl PCR product in the nPCR was the same as above. The nPCR condition involved an initial denaturation at 95 °C for 5 min, followed by 35 cycles of a denaturing step at 94 °C for 1 min, annealing at 55 °C for 1 min for *B. bigemina* and 58 °C for 1 min for *B*. *bovis*, and extension at 72 °C for 1 min and final extension at 72 °C for 7 min. For sequencing, a total of eight PCR-positive samples of *T. orientalis*, five samples of *B. bigemina*, and five samples of *B. bovis* were selected.

**Table 4:** Primer sequences used in this study

Specificity	Primer designation	Essay	Sequence (5’-3’)	Product size	Ref
*T. orientalis*	MPSP	PCR	CTTTGCTCAGGATACTTCCT	776	Ota et al. (2009) [51]
			ACGGCAAGTGGTGAGAACT		
*B. bovis*	SBP2	PCR	CCGAATTCCTGGAAGTGGATCTCATGCAACC	1236	AbouLaila et al. (2010) [52]
			ATCTCGAGTCACGAGCACTCTACGGCTTTGCAG		
		nPCR	CGAATCTAGGCATATAAGGCAT	580	
			ATCCCCTCCTAAGGTTGGCTAC		
*B. bigemina*	BbigRAP-1a	PCR	GAGTCTGCCAAATCCTTAC	879	Terkawi et al. (2011) [2]
			TCCTCTACAGCTGCTTCG		
		nPCR	AGCTTGCTTTCACAACTCGCC	412	
			TTGGTGCTTTGACCGACGACAT		

Ref – reference

Note: Primer sequences used in this study were adapted from previously published protocols by Ota et al. (2009) [48], AbouLaila et al.(2010) [49] and Terkawi et al.(2011) [2]

### DNA sequencing and phylogenetics of *T. orientalis*

After PCR amplification, the expected bands were visualized on a 1.5% agarose gel, excised under UV light, and purified using the QIAquick Gel Extraction Kit (Geneaid Biotech Ltd., New Taipei City, Taipei) according to the manufacturer’s instructions. The purified PCR products were sequenced using both forward and reverse primers at a commercial sequencing facility. The resulting sequences were edited and aligned using BIOEDIT [53], followed by phylogenetic analysis performed in MEGA version 11 [54]. A total of eight *T. orientalis* sequences from this study were deposited in GenBank with accession numbers PV592819–PV592826. Phylogenetic trees were constructed using the Neighbor-Joining method with 1,000 bootstrap replications to assess the reliability of the branching patterns. The Tamura 2-parameter model with gamma distribution and invariant sites (T92 + G + I) was selected as the best-fit substitution model based on the lowest Bayesian Information Criterion (BIC) value. Additionally, genetic distances were estimated using the Jukes–Cantor (JC) model.

### Statistical analysis

All statistical analyses were performed using R statistical software (version 4.1.3) [55] employing the ‘stats’ [55] and ‘rms’ packages [56]. Descriptive statistics were generated, including frequency distributions, tables, and maps, to provide an overview of the data. Univariable and multivariable logistic regression models were applied to evaluate the associations between parasite presence in buffaloes and potential risk factors. For each variable, univariable logistic regression analysis was conducted, calculating the odds ratios (OR) with 95% confidence intervals (CI). Variables with a *p*-value < 0.2 in the univariable analysis were then included in the multivariable logistic regression model. To address potential multicollinearity, categorical risk factors were evaluated using the chi-square test with the ‘gmodels’ package [57]; when identifying multicollinearity (*p* < 0.05), the factor with greater biological relevance was retained. Model selection was guided by the Akaike Information Criterion (AIC), and model fit was assessed with the Hosmer-Lemeshow test [58] applied to test the assumption of goodness-of-fit of the final model. The form of the multivariable logistic regression model was determined using the following equation:

$$\:In\:\left(\frac{{P}_{i}}{1-{P}_{i}}\right)=\:{\beta\:}_{0}+\:{\beta\:}_{1}{X}_{1}+\dots\:+{\beta\:}_{k}{X}_{k}$$

where $\:{P}_{i}$ represents the presence of blood parasite infection status on animal $\:i(i=1,...,155)$, $\:\:{X}_{k}$ denotes a set of risk factors ($\:{X}_{k}=1,...,k$) and $\:{\beta\:}_{k}$ corresponds to the estimated coefficient for each respective risk factor ($\:{\beta\:}_{k}=1,...,k$).

MCA was conducted to examine the associations between variable categories, including (1) the presence of blood parasite infection status (single and co-infection) and (2) the origin of the animals (location, age, BCS, sex and ectoparasite infestation). The analysis was performed using the MCA function from the FactoMineR package [59] in R statistical software. Inertia values were automatically calculated as part of the decomposition of the chi-square (χ²) statistic, a standard output of the MCA function. The Burt matrix was used to represent the relationships between categorical variables. Visualization of the results, including the contributions of each dimension to the total inertia, was performed using the Factoextra package [60].

## Electronic Supplementary Material

Below is the link to the electronic supplementary material.

Supplementary Material 1

## Data Availability

Data is provided within the manuscript or supplementary information files.
