# Supplementary material for: Epidemiology and factors associated with the infection of Babesia bigemina, Babesia bovis, and Theileria orientalis in Thale Noi Wetland buffaloes (Bubalus bubalis), Southern Thailand
Source: BMC Vet Res. 2025 Jun 2;21:397. doi: 10.1186/s12917-025-04846-1 (PMC12128270; doi:10.1186/s12917-025-04846-1)
Supplement: Supplementary file 1 — Supplementary Material 1 [file 12917_2025_4846_MOESM1_ESM.docx]

Questionnaire: Epidemiology and Prevalence of Hemoprotozoan Parasites in Water Buffaloes in Thale Noi Wildlife Non-Hunting Area, Thailand

Instructions: Please help us by answering the following questions truthfully. Fill in the blanks or check the boxes as appropriate.

Date of Survey: ____Village No.: _____ Subdistrict: _____________District: ________

Farm No.: _______ Geographic Coordinates: UTM(E) ________ UTM(N) _________

# Section A: Farmer Demographics

1. Age of respondent: ___________ years

2. Gender of respondent: ☐ Male ☐ Female

3. Experience in buffalo farming: __________ years

4. Education level: ☐ No formal education ☐ Primary school

☐ Secondary school ☐ Bachelor’s degree ☐ Postgraduate

# Section B: Animal Characteristics

1. Sex of the buffalo: ☐ Male ☐ Female

2. Age group: ☐ 1–5 years ☐ More than 5 years

3. Presence of ectoparasites (e.g. ticks, lice): ☐ Yes ☐ No

3.1 If yes, Location of ectoparasites__________________

4. Body condition score (BCS): ☐ Emaciated ☐ Thin ☐ Good

5. Has your farm experienced any buffalo abortions within the last 12 months?

☐ Yes ☐ No

# Section C: Farm Practices

5. Number of buffaloes: ☐ 1–4 animals ☐ 5 or more animals

6. Main purpose of raising buffaloes:

☐ Selling calves for fattening
 ☐ Selling fattened buffaloes for slaughter
 ☐ Selling breeding females
 ☐ Semen sale or breeding bulls
 ☐ Household consumption
 ☐ Others: __________________________

7. Type and management of buffalo farming:

☐ All-time free grazing in Thale Noi
 ☐ Herd mixing with daytime grazing ☐ Stall feeding

8. Buffalo rearing method

☐ Communal herding ☐ Individual rearing

9. Presence of other livestock (e.g. beef cattle)

☐ Yes ☐ No

10. Do you quarantine new animals before introducing them to the herd?

☐ Yes ☐ No

11. Have you received training in buffalo management or ectoparasite control by government agencies?

☐ Yes, latest training: ______________________
 ☐ No

12. Have you seen ectoparasites on your buffaloes? ☐ Yes, type: _______________ ☐ No

13. Have you ever been bitten by ectoparasites from buffaloes? ☐ Yes, type: ___________ ☐ No

14. In your opinion, what are the health impacts of blood-sucking ectoparasites on buffaloes? (Check all that apply)

☐ Anemia
 ☐ Lethargy/loss of appetite
 ☐ Weight loss
 ☐ Fever
 ☐ Abortion
 ☐ Dystocia
 ☐ Hematuria
 ☐ Poor conception
 ☐ Skin lesions
 ☐ No impact

# Section D: Practices in Preventing and Controlling Hemoprotozoan Diseases

| **Question** | **Yes** | **No** | **Comments** |
| --- | --- | --- | --- |
| 1. The farm has a parasite control program for internal and external parasites. |  |  |  |
| 2. External parasites acting as disease vectors are regularly controlled. |  |  |  |
| 3. Tick control program: Use acaricides |  |  |  |
| 4. The frequency of applying acaricides on your farm: Monthly |  |  |  |
| 5. Notification Livestock officers when their animals were sick |  |  |  |
| 6. Livestock officers support acaricides. |  |  |  |
| 7. Without livestock officers support’ you manage the program yourself. |  |  |  |
| 8. You check your body for ticks after handling the tick-infested |  |  |  |
